# Culture-based analysis of *Pristionchus*-associated microbiota from beetles and figs for studying nematode-bacterial interactions

**DOI:** 10.1371/journal.pone.0198018

**Published:** 2018-06-04

**Authors:** Nermin Akduman, Christian Rödelsperger, Ralf J. Sommer

**Affiliations:** Max Planck Institute for Developmental Biology, Department of Evolutionary Biology, Max-Planck-Ring 5, Tübingen, Germany; Universidade de Coimbra, PORTUGAL

## Abstract

The interplay with bacteria is of crucial importance for the interaction of multicellular organisms with their environments. Studying the associations between the nematode model organisms *Caenorhabditis elegans* and *Pristionchus pacificus* with bacteria constitutes a powerful system to investigate these interactions at a mechanistic level. *P*. *pacificus* is found in association with scarab beetles in nature and recent studies revealed the succession and dynamics of this nematode and its microbiome during the decomposition of one particular host species, the rhinoceros beetle *Oryctes borbonicus* on La Réunion Island. However, these studies were performed using culture-free methods, with no attempt made to establish bacterial cultures from the beetle-nematode ecosystem and to investigate the effects of these microbes on life history traits in *P*. *pacificus*. Here, we establish and characterize a collection of 136 bacterial strains that have been isolated from scarab beetles and figs, another *Pristionchus*-associated environment, as a resource for studying their effect on various nematode traits. Classification based on 16S sequencing identified members of four bacterial phyla with the class of Gammaproteobacteria representing the majority with 81 strains. Assessing the survival of *P*. *pacificus* on individual bacteria allowed us to propose candidate groups of pathogens such as Bacillaceae, Actinobacteria, and *Serratia*. In combination with chemoattraction data, it was revealed that *P*. *pacificus* is able to recognize and avoid certain groups of pathogens, but not others. Our collection of bacterial strains forms a natural resource to study the effects of bacterial diet on development and other traits. Furthermore, these results will form the basis of future studies to elucidate the molecular mechanisms of recognition and pathogenicity.

## Introduction

Bacteria form an integral part of the ecology of all living beings and the influence of the gut microbiota on human health has been increasingly recognized during the last decade [1]. Nematodes like *C*. *elegans* are an excellent model to study the interactions between bacteria and their hosts [2], because they are easy to grow using monoxenic bacterial cultures, eg. *Escherichia coli* OP50 as food source. In addition, worms as well as bacteria are genetically tractable, which can provide detailed mechanistic insights into the interaction between host and bacteria and their impact on development and behavior [3,4]. We study the nematode *Pristionchus pacificus* a close relative of the rhabditid *C*. *elegans*, but belonging to the Family Diplogastridae [5]. *P*. *pacificus* and *C*. *elegans* have been estimated to have diverged 280–430 million years ago [6]. *P*. *pacificus* is found in a necromenic association with scarab beetles [7], i.e. nematodes are maintained as growth-arrested dauer larvae on the beetle and upon the beetle’s death resume development and reproduce. They feed on the microorganisms growing on the beetle’s carcass and recent decomposition studies using the rhinoceros beetle *Oryctes borbonicus* from La Réunion Island as host have indicated that the decaying beetles and *P*. *pacificus* have largely overlapping microbiomes [7]. While *P*. *pacificus* and *C*. *elegans* share many biological features, such as the mode of reproduction, the presence of an alternative developmentally arrested dauer stage, and the same chromosome number, nematodes of the *P*. *pacificus* lineage have gained the ability to form tooth-like structures that allow them to predate on other nematodes [8–11]. Interestingly, these feeding structures represent an example of phenotypic plasticity because *P*. *pacificus* can form two alternative mouth forms with stenostomatous animals being strict bacterial feeders, whereas eurystomatous animals are omnivorous feeders that can also kill other nematodes [11]. Whether or not these predatory structures are formed during development is environmentally controlled. Thus, *Pristionchus* mouth-form plasticity represents a developmental decision similar to other examples of phenotypic plasticity in animals, such as the caste system in social insects [12] or color patterns in butterfly wings [13]. To explore the full range of environmental variables that potentially influence developmental decisions, we have recently started to modify culture conditions [14] and tested food sources other than *E*. *coli* OP50 bacteria [15]. Specifically, these studies have shown that growth of worms on yeasts or in liquid culture conditions has an effect on mouth-form plasticity [14,15]. The association of *Pristionchus* nematodes with scarab beetles is stable over millions of years of evolution and has resulted in more than 30 *Pristionchus* species that are found worldwide, often in species-specific interactions with scarab beetles [16]. In addition, a recent study discovered a second branch of the *Pristionchus* genus that is found in association with figs and fig wasps [17]. Strikingly, fig-associated *Pristionchus* species are even capable of producing up to five different mouth morphotypes, whereas beetle associated *Pristionchus* are usually dimorphic [17]. Evidence supported that distinct morphotypes were associated with the degree of maturity of figs and it was hypothesized that the presence of certain bacteria may trigger these developmental decisions. Unfortunately, we failed to cultivate fig-associated *Pristionchus* nematodes permanently under laboratory conditions, which prohibited the further elucidation of environmental cues controlling the development of individual morphs.

The interactions between *Pristionchus* nematodes and bacteria have been studied in the last decade largely by exploring beetle-derived bacteria from Germany and other European sampling sites [18]. These original studies had indicated differences in the response of *P*. *pacificus* and *C*. *elegans* to *Bacillus* ssp., which have initiated large-scale studies of Bacilli and their effect on both nematodes [19–22]. However, *P*. *pacificus* does not normally occur in Europe and the original bacterial isolates mentioned above were obtained from *P*. *maupasi* and *P*. *entomophagus*.

To further study the interactions between *P*. *pacificus* with its natural bacteria, we have carried out a culture-based approach to isolate and investigate nematode associated bacteria from three different locations in Asia, Africa and the Indian Ocean and used both hosts, beetles and figs. Specifically, we isolated bacteria from figs retrieved from Vietnam, South Africa, and La Réunion Island and from scarab beetles from La Réunion Island, which forms a hotspot of *P*. *pacificus* diversity [7,23]. In total, we classified 136 bacterial isolates based on their 16S ribosomal RNA sequences. We test the nematodes´ capability to survive on these bacteria and their chemoattractive potential relative to standard *E*. *coli* OP50 cultures. Our data show that most of the isolated bacteria support growth of *P*. *pacificus* worms and most bacteria seem to be more attractive than the standard *E*. *coli* OP50 strain. Furthermore, the finding of a weak correlation between survival and chemotaxis data raises the question to what extent nematodes can sense which food sources are suitable for them.

## Materials and methods

### Nematode and bacterial culture conditions

The wild type strain of *P*. *pacificus* (PS312) was grown at 20°C on nematode growth medium (NGM) seeded with *E*. *coli* OP50 before use in experiments. Bacterial strains were cultured on following growth media: LB agar (1% tryptone, 0.5% yeast extract, 1% NaCl and 1.5% agar), NGM [24], YPD agar (2% bacteriological peptone, 1% Yeast extract, 2% Glucose and 1.5% Agar), NA (Thermo scientific, Oxoid, CM0003), TSA (1.5% tryptone, 0.5% soytone –enzymatic digest of soybean meal, 0.5% sodium chloride, 1.5% agar), PDA (Difco™ Potato Dextrose Media, BD, 1.5% Agar).

### Sample collection and isolation of wild bacteria

**Fig 1:**
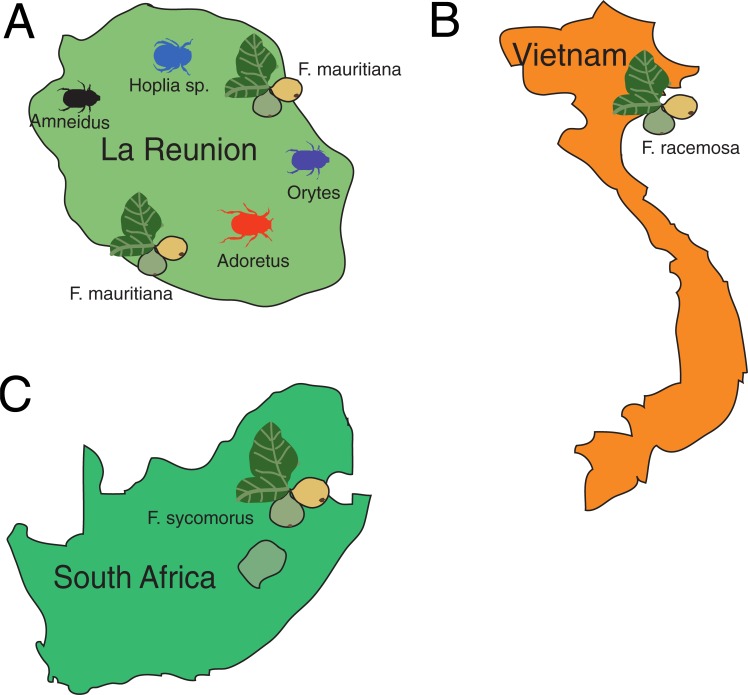
Regional maps of beetle and fig sampling sites. (A) Map of La Réunion Island showing the approximate beetle and fig sampling sites. *O*. *borbonicus*, *Adoretus* sp., *Hoplia sp*. and *A*. *godefroyi* beetles were collected to isolate *Pristionchus*-associated bacteria. Similarly, *F*. *mauritiana* figs were sampled and processed for bacteria isolation. (B) and (C) *F*. *sycomorus* (Brummeria, Pretoria, South Africa) and *F*. *racemosa* (Hanoi, Vietnam) figs were dissected under sterile conditions from *Pristionchus*-positive specimens to isolate bacteria.

We collected different beetle species (*Oryctes borbonicus*, *Adoretus* sp., *Hoplia sp*. and *Amneidus* godefroyi) from La Réunion Island (Fig 1A) using sweeping nets, black light traps and pitfall traps baited with dung [25]. Only adult beetles were collected before being transferred to the laboratory alive. To avoid contamination by human associated bacteria, all sample collections were done wearing gloves. Under sterile conditions animals were sacrificed by cutting them in half transversely and all body parts were placed on LB agar plates. Bacteria were only isolated from beetles that also showed the presence of *Pristionchus* nematodes. Plates that were negative for *Pristionchus* but that were positive for other nematodes were discarded. Isolated bacteria were spotted on LB plates and colonies were singled out for two rounds to get pure bacterial strains. For genotyping, bacterial strains were sub-cultured and then prepared for sequencing using PCR amplification of 16S ribosomal RNA genes. Permits for beetle samplings on La Réunion Island were provided by Sylvain Leonard from the Office National des Forets and Benoit Lequette from the Parc National de La Réunion between 2012 and 2017. Note that the research permits did not allow the disclosure of the exact sampling localities because several beetle species are endangered (i.e. *Oryctes borbonicus*).

To isolate bacteria from figs, we collected several fig species including *F*. *mauritiana* (La Réunion), *F*. *sycomorus* (South Africa), and *F*. *racemosa* (Vietnam) (Fig 1B and 1C). Individual figs were dissected under sterile conditions and the presence of *Pristionchus* nematodes was confirmed. 500 μl of fig juice was extracted with a sterile pipette and suspended in sterile PBS, and aliquots were spread on LB, NGM, YPD, NA, TSA, and PDA agar plates and then grown for 1–2 d at 30°C. Single colonies were isolated from plates, grown in LB (shaking at 180 rpm, 30°C) or until significant growth was achieved, and frozen at −80°C in 25% glycerol stocks. Permits for fig samplings on La Réunion Island were provided by Benoit Lequette from the Parc National de La Réunion between 2014 and 2016.

### Bacteria identification

Each bacterial colony was grown overnight in LB broth and DNA was extracted using Epicenter MasterPure DNA purification kit (Illumina, San Diego, USA). Polymerase chain reaction (PCR) amplification of bacterial 16S rRNA genes was carried out in 25 μl reactions using a universal primer set SSU 27f (5’-AGAGTTTGATCMTGGCTCAG-3’) and SSU 1492r (5’-TACGGYTACCTTGTTACGACTT-3’) [18]. Thermal cycling conditions were as follows: 3 min at 95°C followed by 30 cycles of 15 s at 95°C, 30 s at 55°C, 1.5 min at 72°C, and a final step of 8 min at 72°C. A typical reaction contained 2 μl 10x PCR buffer, 2 μl 2·mmol·l^–1^ dNTPs, 1μl 10 μmol·l^–1^ 27f, 1μL10 μmol·l^–1^ 1492r, one unit of Taq DNA polymerase, 12.8·μl H_2_O and 1μL of bacterial DNA. PCR amplicons were visualized by standard agarose gel electrophoresis [26]. All high quality 16S rRNA gene sequences of bacteria were classified by the SILVAngs webservice of the SILVA database [27].

### Survival assays

Bacterial liquid cultures were established by inoculating 5ml LB with a single bacterial colony. Subsequently, cultures were grown overnight at 30°C. Bacterial suspensions (50μl) were spread with an L-shaped spreader on NGM medium petri dishes with diameter of 6cm and were incubated overnight. Twenty young adult *P*. *pacificus* worms that were well fed on *E*. *coli* OP50, were washed five times with PBS and picked to intermediate plates seeded with test bacteria to reduce contamination, a standard procedure in nematode survival assays. One hour later, worms were picked to the final assay plates seeded with test bacteria. Each plate was kept at 20°C. Survival of worms was monitored daily for 5 days. Nematodes were transferred every two days to fresh plates to prevent misidentification of original worms from offspring. Mortality was determined by prodding worms with a metal pick and nematodes that did not respond were considered dead. In total, we performed three biological replicates per bacterial strain.

### Chemotaxis assays

Chemotaxis assays were modified from previous studies [28,29]. Briefly, 20 μl of overnight bacterial suspension was placed 0.5 cm away from the edge of a 6 cm Petri dish filled with NGM medium. The same amount of *E*. *coli* OP50 was placed on the opposing side acting as the counter attractant. Approximately 50–200 J4/adult stage *P*. *pacificus* individuals were placed at the edge of the plate, equidistant to each of the bacterial spots. All nematodes were previously fed on *E*. *coli* OP50. Plates were incubated at room temperature. After 3h the number of nematodes found in each bacterial spot was recorded. A chemotaxis index was used to score the response of the nematodes, which consisted of: number of nematodes in the region spotted with test bacteria minus the number of nematodes in control bacteria spots, the result was divided by the total number of nematodes counted [29]. This gave a chemotaxis score ranging from –1.0 (repulsion) to 1.0 (attraction). Three plates were used per replicate, and the procedure was repeated four to six times for each bacterium.

### Statistical analysis

For each bacteria analysed, we averaged the survival and chemotaxis values from all replicates and employed a Wilcoxon rank-sum test to test for significant differences between taxonomic groups. P-values were corrected for multiple testing with the Bonferroni method. Correlation between survival and chemotaxis data was calculated as Spearman correlation. All analyses and plots were done using the statistical program R.

## Results

### A bacterial strain collection from *Pristionchus*-associated environments

**Fig 2:**
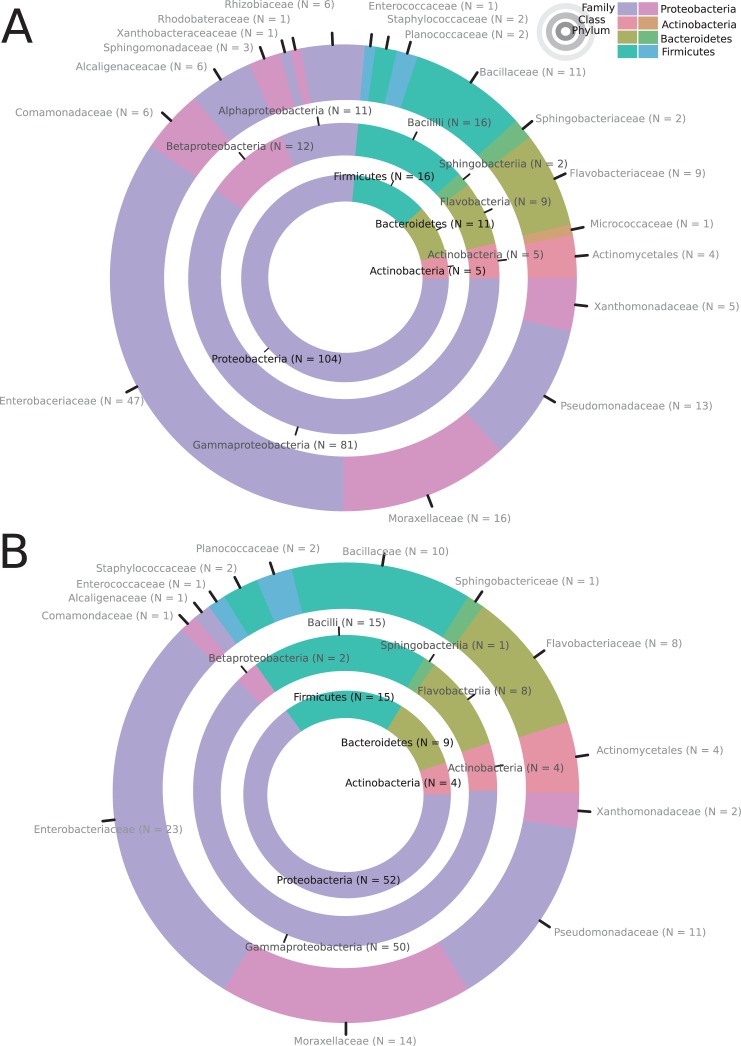
Taxonomic distribution of bacterial isolates. (A) Circles show the distribution of bacterial strains at the level of phyla (innermost circle), class (middle circle), and family (outermost circle) based on classification of the 16S ribosomal RNA gene [27]. Proteobacteria are by far the largest group (N = 104 strains). (B) Distribution of bacterial strains that were isolated from beetles on La Réunion Island.

We used fig and beetle samples provided by our collaborators, to isolate and cultivate a total of 136 bacterial strains (S1 Table). Specifically, 80 different bacterial strains were isolated from beetle species (*O*. *borbonicus*, *Adoretus* sp., *Hoplia sp*. and *A*. *godefroyi* collected from La Réunion Island) (Fig 1A). In addition, we isolated 26 bacterial strains from *F*. *mauritiana* (La Réunion), 21 strains from *F*. *racemosa* (Vietnam) and nine strains from *F*. *sycomorus* (South Africa)(Fig 1A). Each strain was classified after sequencing a fragment of the 16S ribosomal RNA gene. Overall, we isolated strains belonging to four bacterial phyla, Proteobacteria, Firmicutes, Bacteriodetes, and Actinobacteria (Fig 2A). The same four phyla were also present when only considering bacterial isolates from beetles (Fig 2B). These findings are consistent with the recent high throughput sequencing of the microbiome of *O*. *borbonicus* and *P*. *pacificus* [7]. Out of the 104 isolated strains of Proteobacteria, 47 are representatives of the family Enterobaceriaceae, which has also been found to be by far the most abundant family of bacteria in decaying beetles [7]. Thus, our culture-based method is largely consistent with the culture-free results of *O*. *borbonicus*-associated microbes and provides a collection of 136 bacterial strains for laboratory studies.

### Most bacterial isolates are not pathogenic to *P*. *pacificus*

**Fig 3:**
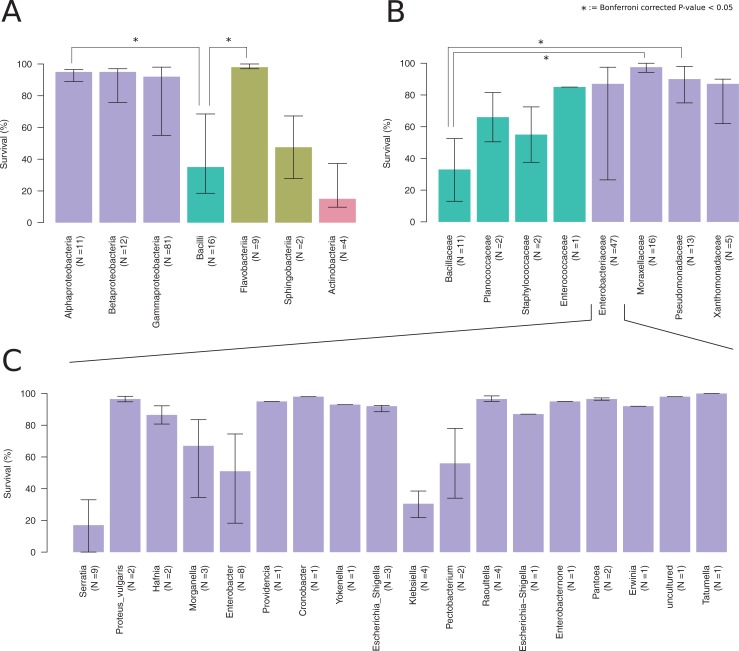
Survival of *P*. *pacificus* nematodes on different bacteria. (A) Bars show median and interquartile ranges of survival of *P*. *pacificus* worms in response to various bacterial classes. All pairwise tests for significantly different survival were done by a Wilcoxon rank-sum test. Results that remained significant after Bonferroni correction are highlighted. (B) Distribution of survival rate for deeply sampled and highly variable bacterial classes (Bacilli and Gammaproteobacteria) at higher phylogenetic resolution (family level). Within the class Bacilli, decreased survival is mostly due to strains of the family Bacillaceae. Within the class Gammaproteobacteria, the largest variability in nematode survival is observed in the family Enterobacteriaceae. (C) Distribution of survival within Enterobacteriaceae at genus level.

Next, we tested how well *P*. *pacificus* strains can survive on the isolated bacteria. To this end, we exposed 20 young adults to individual bacterial strains and counted the number of surviving worms after five days. Control experiments performed on *E*. *coli* OP50 showed a survival rate of between 95–100%. In our experimental setup, worms could survive on most of the tested bacteria (Fig 3). Fig 3 shows the result of the survival tests at the level of bacterial classes and selected families. Survival on Bacilli strains was significantly lower (Wilcoxon Rank-Sum test, Bonferroni corrected p-value < 0.05) than on Flavobacteria and Alphaproteobacteria (Fig 3A). In contrast, sample sizes of Actinobacteria and Sphingobacteria were too low to reveal statistically significant differences in nematode survival in comparison to other bacterial classes. However, investigating the survival patterns at higher taxonomic resolution, we found that the lower survival on Bacilli strains is largely due to members of the family Bacillaceae (Fig 3B) and the high variability in Gammaproteobacteria can be attributed to variability in the family Enterobacteriaceae. At the level of genera, the variability within Enterobacteriaceae appears to be caused by lower survival on individual strains belonging to *Serratia*, *Morganella*, *Enterobacter*, *Klebsiella* and *Pectobacterium* (results were not statistically significant after multiple testing correction, Fig 3C). While some *Serratia* strains have previously been described to be pathogenic to *P*. *pacificus* [30], it also seems that some *Enterobacter* and the human pathogen *Klebsiella* strains can also be pathogenic to *P*. *pacificus*.

### *P*. *pacificus* nematodes are attracted to most bacterial isolates

**Fig 4:**
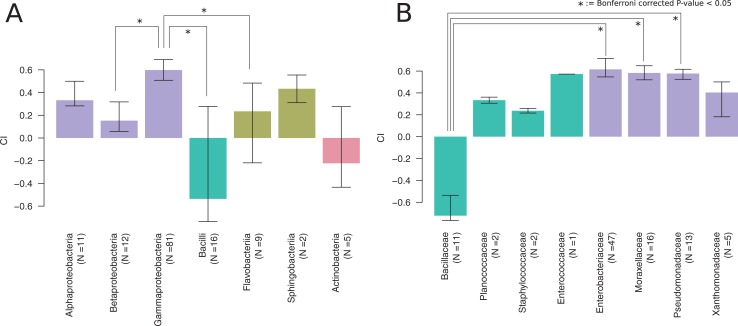
Chemoattraction towards different bacteria. (A) While most bacterial strains seem to be preferred over control spots, the class Bacilli shows a significantly repulsive effect (Wilcoxon test, Bonferroni corrected p-value<0.05) in comparison to three other bacterial classes. (B) Within the class Bacilli, the repulsive effect is largely due to strains of the family Bacillaceae.

To test whether *P*. *pacificus* nematodes are attracted towards the isolated bacteria, we performed chemotaxis assays by giving worms the choice between two alternate food sources. Specifically, we used one spot of the target bacteria opposite one spot of an equal volume *E*. *coli* OP50 and counted the number of worms in each of the spots after three hours. Subsequently, we then calculated a chemotaxis index (CI). A CI of -1 indicates repulsion from the test bacterium, whereas a CI of 1 indicates attraction. Our results show that most bacterial isolates are preferred by *P*. *pacificus* as opposed to control spots. However, individual strains of the classes Actinobacteria, Flavobacteria, and Bacilli showed negative CIs (Fig 4A). Within Bacilli, the repulsive effect was mostly due to the family of Bacillaceae (Fig 4B), whereas other families of Bacilli frequently showed positive CIs indicating strong strain specificity.

### Weak correlation between survival and chemotaxis

**Fig 5:**
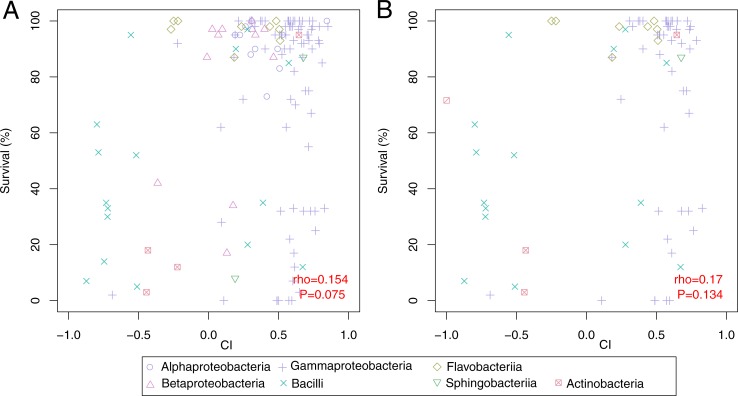
Correlation between survival and chemotaxis. (A) Testing for the correlation between survival and chemotaxis data we found a weak trend (Spearman’s rho = 0.154, P = 0.075) for bacteria resulting in higher survival rates to also have higher chemotaxis indices, compared to strains that do not support growth or are pathogenic. (B) Similar correlation tests for those bacterial strains isolated from beetles where again, no strong signal was observed (Spearman’s rho = 0.17, P = 0.134).

Combining the results of the survival and chemotaxis assays it appears as if *P*. *pacificus* can recognize and escape from certain pathogens. For example, certain strains of Bacillus and Actinobacteria show low survival of *P*. *pacificus* and strong repulsion in chemoattraction assays (Fig 5). In addition, most non-pathogenic strains seem to be preferred by worms over *E*. *coli* OP50 control spots. To test to what extent *P*. *pacificus* can distinguish suitable food sources from pathogens, we calculated the correlation coefficients between chemotaxis and survival data (Fig 5A), which revealed only a weak trend for the whole data set (Spearman’s rho = 0.154, P = 0.075). Even restricting the analysis to bacterial strains that were isolated from beetles and are therefore more likely to be seen by *P*. *pacificus* worms in the wild did not result in a higher correlation (Fig 5B). In particular, *P*. *pacificus* is attracted to multiple strains of the genus *Serratia*, which are known pathogens of this nematode species (Figs 3C and 4). Thus, *P*. *pacificus* can recognize and avoid certain but not all pathogens.

## Discussion

In this study we have isolated and characterized 136 bacterial strains from *Pristionchus-*associated environments, scarab beetles and figs. As ecologically relevant results of microbial-animal interactions are most likely to be obtained when microbes from the same environment are used in which the test organism lives, we only isolated bacteria from samples that showed the presence of *Pristionchus* nematodes. Despite the fact that our culture conditions most likely only allow isolation of a small percentage of the total bacterial community, the cultivable strains will form a powerful resource to study how *Pristionchus* nematodes interact with their environment and in particular, how bacterial diet can influence developmental decisions, such as the mouth form dimorphism [9]. Previous work on *Cryptococcus* yeast has demonstrated shifts in mouth form ratios of *P*. *pacificus* nematodes upon altered diet [15]. Thus, it is highly likely that some of the isolated bacteria induce similar effects.

We have screened *P*. *pacificus* survival on all isolated bacterial strains and found that multiple strains of diverse taxonomic groups are candidates for nematode pathogens. Among these, the genus *Serratia* has been previously described as potent killer of *P*. *pacificus* and *C*. *elegans* [30] and our survival assays showed that one of the *Stenotrophomonas* sp. isolates can be a potential pathogen to *P*. *pacificus*. Note that survival of nematodes on individual bacteria does not necessarily indicate the ability of the nematode to grow and reproduce on these strains. However, during the course of experiments, we kept nematodes on various bacterial strains for several generations indicating that *P*. *pacificus* can complete its life cycle on many of the isolated bacteria. It is important to note that in nature *P*. *pacificus* is exposed to a mixture of bacteria and therefore, survival assays performed with monoxenic cultures of test bacteria are partially artificial. Future studies will aim to study combinations of bacteria simultaneously, thereby mimicking more closely the situation seen in nature.

Complementary experiments of chemotaxis showed that bacterial classes like Bacilli and Actinobacteria that caused reduced survival are avoided by *P*. *pacificus*, which preferred feeding on *E*. *coli* OP50 when having the choice. However, explicitly testing for a correlation between survival and chemotaxis data did not allow the conclusion that *P*. *pacificus* can broadly recognize and avoid pathogenic bacteria. Overall, our chemotaxis experiments may suggest that most isolates are preferred over *E*. *coli* OP50 control spots. This finding came as a surprise given that the strain PS312 is in permanent culture since 1988 and has been exclusively fed on *E*. *coli* OP50 [5], but apparently has not developed a preference for it. Nevertheless this finding is consistent with observations from *C*. *elegans* showing that other bacteria such as *Comamonas* are much better food sources than *E*. *coli* OP50 [5,31]. However, the statement that *P*. *pacificus* prefers many bacteria over *E*. *coli* OP50 is to be regarded with care because the assay conditions utilized can not control for the exact bacterial concentration. In many of the assays, we observed that thicker colonies are not necessarily preferred by nematodes, suggesting that even if differences in bacterial concentrations exist, they seem to have a minor effect on the results of the chemotaxis assays. This might be due to the strong *P*. *pacificus* perception of oxygen [32]. Taken together, the survival and chemotaxis data showed substantial phylogenetic signal indicating that related bacteria give rise to a similar response in terms of nematode survival and chemoattraction. This may suggest that the overall biochemical composition of bacteria causes the observed effect on *P*. *pacificus* nematodes. Interestingly both potential groups of pathogens (Bacilli and Actinobacteria) that can be recognized and avoided by *P*. *pacificus* are Gram-positive and spore forming bacteria suggesting that one or multiple features associated or correlated with the Gram-positive life style and/or spore formation are responsible for the response in nematodes.

In summary, the collection of bacterial strains that has been described in this study constitutes a resource for future studies of interactions between nematodes and bacteria. Our findings raise a number of interesting questions for future investigations, e.g. which bacterial factors are recognized by worms and how do they sense them? Given the substantial variability in survival, how are these patterns reflected in terms of development and other life history traits? Which of the isolated bacteria is the best food source for *P*. *pacificus*? Given that these associations can be studied in very controlled conditions and nematodes and bacteria are genetically tractable, combined investigation of nematodes and bacteria forms a powerful experimental system to study the effect of microbiota on organisms at a mechanistic level.

## Supporting information

S1 Table *Pristionchus*-associated bacteria strain names and sequence information. (XLSX)
